# GPVI inhibition: Advancing antithrombotic therapy in cardiovascular disease

**DOI:** 10.1093/ehjcvp/pvae018

**Published:** 2024-03-07

**Authors:** Alexandre Slater, Sophia Khattak, Mark R Thomas

**Affiliations:** Institute of Cardiovascular Sciences, College of Medical and Dental Sciences, University of Birmingham, B15 2TT, Birmingham, UK; Institute of Cardiovascular Sciences, College of Medical and Dental Sciences, University of Birmingham, B15 2TT, Birmingham, UK; Cardiology Department, Queen Elizabeth Hospital, University Hospitals Birmingham, B15 2GW, Birmingham, UK; Institute of Cardiovascular Sciences, College of Medical and Dental Sciences, University of Birmingham, B15 2TT, Birmingham, UK; Cardiology Department, Queen Elizabeth Hospital, University Hospitals Birmingham, B15 2GW, Birmingham, UK

**Keywords:** Glycoprotein VI, Arterial thrombosis, Glenzocimab, Revacept, Anti-platelet

## Abstract

Glycoprotein (GP) VI (GPVI) plays a major role in thrombosis but not haemostasis, making it a promising antithrombotic target. The primary role of GPVI on the surface of platelets is a signalling receptor for collagen, which is one of the most potent thrombotic sub-endothelial components that is exposed by atherosclerotic plaque rupture. Inhibition of GPVI has therefore been investigated as a strategy for treatment and prevention of atherothrombosis, such as during stroke and acute coronary syndromes. A range of specific GPVI inhibitors have been characterized, and two of these inhibitors, glenzocimab and revacept, have completed Phase II clinical trials in ischaemic stroke. In this review, we summarize mechanisms of GPVI activation and the latest progress of clinically tested GPVI inhibitors, including their mechanisms of action. By focusing on what is known about GPVI activation, we also discuss whether alternate strategies could be used to target GPVI.

## Introduction

Platelets are small anucleate cells that play a pivotal role in both haemostasis and thrombosis.^1,2^ Platelets have been widely targeted for treating thrombotic disorders, and the most common clinically used antiplatelet drugs include aspirin (an inhibitor of cyclooxygenase-1^3^), platelet P2Y_12_ receptor antagonists,^4^ and integrin Glycoprotein (GP) IIb/IIIa (GPIIb/IIIa) inhibitors.^5–7^ Dual antiplatelet therapy consisting of aspirin and a P2Y_12_ inhibitor is used routinely for the treatment of acute coronary syndrome, and P2Y_12_ inhibitors have also shown benefits in a wide range of atherothrombotic conditions, such as stroke and peripheral arterial disease. Despite the significant benefits these therapies have had for patients with atherothrombosis, they are all associated with a significantly increased risk of major bleeding due to their roles in normal haemostatic function. There is currently an unmet clinical need to find targets on platelets that drive thrombosis without affecting normal haemostatic function and increasing the risk of bleeding.

## Glycoprotein VI as a safe antithrombotic target

The critical role of platelet receptor glycoprotein VI (GPVI) in thrombus formation is well established. GPVI is strongly activated by atherosclerotic plaque due to the high concentration of collagen I and III.^8^ Atherosclerotic plaque rupture is a key driver of diseases such as stroke and myocardial infarction^9,10^ and exposes sub-endothelial collagen, which activates platelets specifically through GPVI.^8,11,12^ The specific involvement of GPVI in platelet activation by plaque but not normal haemostatic function has led to it being an attractive target for treating arterial thrombosis.^13,14^

The platelets from GPVI-deficient mice do not tether to the sub-endothelium of injured artery walls,^15^ have reduced thrombus formation on collagen fibrils,^16^ are protected from thromboembolism,^17^ and protect mice from ferric chloride-induced thrombosis.^18^ These mice showed no increased bleeding times, suggesting that, in mice, GPVI does not play a major role in haemostasis. GPVI-deficient patients have also been reported in the Chilean population.^19^ These patients have a single nucleotide insertion that results in a truncated extracellular GPVI, which is retained in the cytosol and cannot be presented at the membrane. These patients only presented with, at most, mild bleeding disorders. Furthermore, relatives heterozygous for this mutation showed no risk of bleeding.^19^ This is also consistent with data from heterozygous GPVI-deficient mice, which showed no effect on tail bleeding time.^17^ GPVI-deficient platelets from patients were able to adhere to collagen, but no phosphatidyl serine exposure was observed.^20^ Preclinical studies have mostly focused on arterial thrombosis and not venous thrombosis. This is largely due to the reduced involvement of collagen-induced platelet activation in venous thrombosis, but there is emerging evidence of GPVI also playing a role in this disease,^21^ which may warrant further investigation. Although bleeding defects have been reported in GPVI-deficient patients (reviewed by Arthur *et al*.^22^), it is worth noting that many of these patients may have additional mutations of other receptors. In particular, many of the patients in these reports have a coexistent thrombocytopenia that is not caused by GPVI deficiency but may be the actual underlying cause of the bleeding defect. Targeting this receptor was therefore not believed to lead to unacceptable effects on haemostasis and has warranted further study.^23^ In keeping with this, in a recently published clinical trial of the GPVI inhibitor glenzocimab in stroke, glenzocimab was not associated with higher rates of intracranial haemorrhage than placebo.^24^

## Mechanisms of GPVI activation

Understanding the structure and mechanisms of GPVI activation is critical for the development of specific inhibitors. GPVI activation is a well-studied area, but the molecular mechanisms involved are relatively poorly understood. The only known structures of GPVI are of the extracellular D1 and D2 domains, and the majority of biochemical and biophysical assays utilize recombinant D1 + D2. It is therefore not surprising, as the most well characterized region, that all known direct GPVI inhibitors target these domains. D1 and D2 are responsible for ligand binding, and inhibiting ligand binding is an effective way to inhibit receptor function. However, there may be other regions of GPVI that are critical for GPVI activation that can be targeted with greater effectiveness. A summary of what is known about GPVI structure and how this relates to function is discussed below.

GPVI was first identified as a band on a Two-dimensional (2D),^25^ and its link with collagen was documented 5 years later by studying patients with platelets that did not respond to collagen.^26,27^ It was later described as the major collagen receptor on platelets.^28,29^ Human GPVI is a 319 amino acid Type I transmembrane protein in the Immunoglobulin (Ig)-like superfamily of receptors.^30^ It consists of extracellular Ig-like domains, D1 and D2, a glycosylated stalk, single transmembrane region, and short intracellular tail.^31^ GPVI is complexed to the FcR-γ chain in the membrane^32,33^ through a salt bridge formed in the transmembrane regions and interactions within the intracellular tail.^34–36^ The FcR-γ chain is a homodimer, and each chain contains an immunoreceptor tyrosine-based activation motif (ITAM). Cross-linking GPVI induces phosphorylation of the conserved ITAM tyrosine residues by the Src family kinases (SFKs) Fyn, Lyn, and Src.^32,37,38^ Recruitment of Syk to the phosphorylated ITAM initiates downstream signalling that results in activation of PLCγ2 and platelet aggregation.^39–41^

GPVI binds collagen via its extracellular D1 domain.^42–44^ GPVI binds to glycine–proline–hydroxyproline (GPO) repeat motifs found in collagen. This repeated GPO motif is the basis of the synthetic collagen-related peptide (CRP), which is a potent activator of platelets through GPVI,^45–47^ and its exact binding site has been mapped by X-ray crystallography.^44^ Additional GPVI ligands have been identified including the snake venom convulxin,^48,49^ fibrin,^50–53^ and galectin-9.^54^ These ligands have not been mapped, but competition studies have identified that they also bind to the D1 domain.^52,54^ All known activators of GPVI are oligomeric, which highlights GPVI clustering as a key mechanism for activation. The importance of GPVI clustering is highlighted by recent studies showing inhibitory nanobodies specific to GPVI can activate platelets when they are multimerized.^55,56^ Fibrinogen, which is a monovalent ligand for GPVI, only activates GPVI when it is immobilized onto a surface^57^ where it mimics a multivalent surface and can cluster GPVI. GPVI clustering can clearly be visualized by microscopy when platelets are spread on a collagen surface,^58,59^ but how clustering leads to activation remains unknown. Possible mechanisms are that clustering induces a conformational change in GPVI that promotes signalling, GPVI clusters prevent access of regulatory phosphatases, and GPVI is always active, but clustering is required to amplify the signal resulting in sustained signalling. Further studies are required of GPVI clusters to deduce the mechanisms of action.

GPVI is known to dimerize, although significant advancements have been made in recent years that have questioned the role of dimerization in GPVI activation. The idea of GPVI dimerization was first proposed when dimeric GPVI-specific antibody JAQ1 was shown to induce low levels of GPVI signalling.^60^ Later studies by Miura *et al*.^61^ revealed recombinant GPVI only had affinity for collagen when expressed in a dimeric form (through fusing GPVI to the Fc domain of IgG). The first crystal structure of the GPVI D1 and D2 domains revealed a back-to-back dimer conformation formed by the interactions of a β-strand from one D2 domain packing against the same strand from an adjacent D2 domain. This conformation has been observed in additional crystal structures.^44^ Extracellular GPVI does not dimerize in solution, suggesting the dimer interface at D2 is weak.^62^ Additional sites of dimerization have been reported within the intracellular tail through the form of disulfide bonds.^63^ The structure of GPVI in complex with an inhibitory nanobody revealed a novel dimeric conformation^64^ that involved a D2-induced domain swap. The D2 hinge region involved in the domain swap was shown to be critical for GPVI signalling, suggesting this conformation could be a biologically active dimer; however, this does not rule out the possibility that GPVI exists in multiple dimeric conformations. A mixture of monomeric and dimeric forms of GPVI have been observed on the cell surface through dimeric GPVI-specific antibodies^65–67^ and advanced microscopy.^68^

The early accepted model for GPVI dimerization was that it induced a conformational change required for ligand binding. This would then lead to further clustering of GPVI dimers, suggesting that inhibiting GPVI dimerization could be an effective way of preventing signalling. However, the concept of GPVI dimers being required for ligand binding was complicated when multiple groups published interaction studies of GPVI with fibrin, with some reporting fibrin bound specifically to dimeric GPVI^51,69^ and others monomeric GPVI.^50,52,70^ The contradicting results were likely due to discrepancies between GPVI constructs, fibrin generation methods, and GPVI detection methods.^71^ A more recent study showed that both monomeric GPVI and dimeric GPVI bind to fibrinogen and the increased binding of dimeric GPVI is due to avidity and not a conformational change.^53,72^ This directly opposed the idea of dimeric GPVI having a specific conformation that is required for ligand binding. This was further supported by a monomeric crystal structure of GPVI that had no significant conformational changes in the ligand-binding domain compared with the dimeric crystal structures.^73^ The crystal structure of GPVI bound to CRP also revealed that the orientation of the binding sites does not support the simultaneous binding of two collagen fibres to one GPVI dimer.^44^ Taking all these observations into account suggests that the role of GPVI dimerization in ligand binding is not essential. A new model proposed that dimerization is not required for ligand binding but is important for increasing the cluster size of GPVI to a threshold required for sustained signalling.^74^ It is clear that GPVI dimers do exist, but their role in receptor function is not fully understood. Specific inhibitors of GPVI dimerization are required to determine its functional role, although this will first require an increased knowledge of how GPVI dimerizes in the membrane.

## GPVI inhibitors

Inhibitors of downstream signalling proteins, including Syk and Btk, are effective at inhibiting GPVI signalling.^75–78^ However, these proteins are involved in a range of signalling pathways on a range of cell types and may lead to more side effects than if GPVI was targeted directly. This review therefore focuses on targeting the GPVI receptor directly. A wide range of GPVI inhibitors have been developed, and two of these have been taken through to clinical trials and will be discussed in detail below. Furthermore, later sections cover additional avenues that can be explored and taken advantage of to develop novel GPVI inhibitors.

### Glenzocimab

Glenzocimab (also known as ACT017) is a humanized version of the GPVI-specific Fab fragment 9O12.^79^ This Fab fragment was raised against the extracellular domains of GPVI and has a binding affinity between 1 and 13 nM.^80^ Glenzocimab disrupts the interaction of GPVI with collagen and thrombus formation under arterial flow conditions.^81^ It was also shown to inhibit GPVI function without affecting bleeding time or platelet count in animal models. In combination with aspirin and ticagrelor, glenzocimab reduced atherosclerotic plaque-induced platelet aggregation by over 95%, sharing antithrombotic effects with GPIIb/IIIa inhibitors but with less apparent effect on markers of general haemostasis.^82^ Atherosclerotic plaque that is exposed after plaque rupture in humans causes platelet activation and occlusive thrombus formation in arteries, leading to myocardial infarction and stroke. Inhibition of this mechanism by glenzocimab has therefore been further investigated as a treatment for atherothrombosis.^83^ A Phase I clinical trial showed that doses of 62.5–2000 mg of glenzocimab were well tolerated in human subjects, with no change in bleeding times, platelet count, or GPVI expression levels.^84^ The calculated terminal half-life of glenzocimab was 10.2 h, and a 1000 mg dose was shown to have maximum inhibitory effect.^85^ A Phase 1b/2a clinical trial, ACTIMIS (NCT03803007), on 60 patients with ischaemic stroke dosed with 1000 mg of glenzocimab or placebo via intravenous infusion was conducted to assess the safety of glenzocimab as an add-on to standard care (thrombolysis ± thrombectomy). Glenzocimab was deemed to be safe, and it was also reported to be associated with a numeric reduction in intracerebral haemorrhage and patient mortality.^24^ The LIBERATE clinical trial has recently started recruiting and is investigating the benefit of glenzocimab vs. placebo in addition to standard care for patients with ST-elevation myocardial infarction (ISRCTN15443962). This study will demonstrate whether glenzocimab reduces myocardial infarct size as assessed by cardiac magnetic resonance imaging (MRI). Glenzocimab has also been tested in patients with COVID-19 in a Phase II study in patients with COVID-19-related acute respiratory distress syndrome, as thrombosis plays an important role in the pathophysiology of COVID-19. Although no safety concerns were reported, the final results are not yet published.

**Figure 1:**
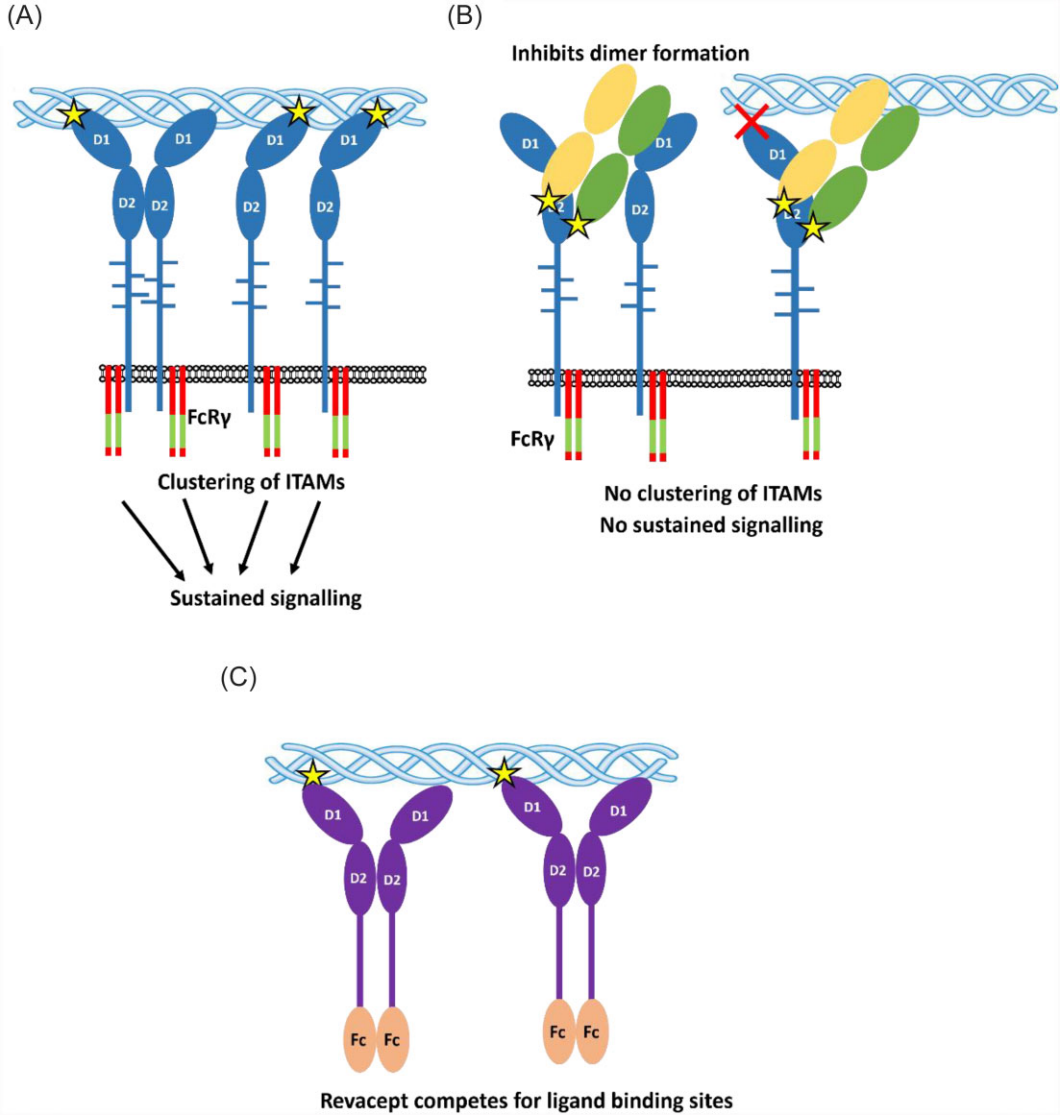
GPVI inhibition by glenzocimab and revacept. (*A*) Clustering of GPVI monomers and dimers by multivalent ligands, such as collagen, results in sustained signalling and platelet activation. The FcR-γ chain associates with GPVI and contains the ITAM sequences that are critical for phosphorylation-induced signalling. Figure adapted from Slater and Jandrot-Perrus.^74^ (*B*) Glenzocimab binds to the D2 domain of GPVI and blocks dimerization and ligand binding. This results in potent inhibition of receptor clustering. (*C*) Revacept mimics extracellular GPVI and competes with the membrane-bound GPVI for ligand binding. GPVI, glycoprotein VI; ITAM, immunoreceptor tyrosine-based activation motif.

In attempts to determine the mechanisms of glenzocimab inhibition, the binding site was mapped to GPVI by X-ray crystallography.^80^ Unexpectedly, despite blocking collagen binding to D1, glenzocimab was mapped to the D2 domain of GPVI. Glenzocimab is currently the only known binder of GPVI that interacts directly with the D2 domain. This makes it not only an effective GPVI inhibitor but also a useful tool for the study of GPVI function. The orientation of the binding site means that despite not interacting with the D1 domain directly, the light chain lies directly in the way of the channel where collagen would bind to D1. Therefore, glenzocimab sterically occludes collagen binding. In addition, the crystal structure showed the binding of glenzocimab interfered with the D2 dimer interface. This was confirmed by fluorescence correlation spectroscopy where dimer formation was fully inhibited. This highlighted the dual action of glenzocimab where it not only sterically occludes collagen binding but also inhibits the formation of dimers. The impact of dimer inhibition is currently not clear as the role of dimer formation is not well understood, as described earlier. However, when thinking about the impact on GPVI cluster size, glenzocimab is affecting both ligand-induced clustering and clustering by dimer formations (*Figure 1B*). The binding to the D2 domain effectively blocks an entire face of D1 from binding to larger ligands due to steric clashes. This increases the chances that glenzocimab will inhibit multiple ligands and not specifically collagen. This is supported by the fact that glenzocimab also inhibits fibrinogen and fibrin binding.^57,82^ This is beneficial as GPVI binding to collagen is believed to be important for the development of the core of a thrombus whereas binding to fibrinogen is more involved in the shell.^86^ Therefore, glenzocimab can inhibit the formation of both the core and shell of a thrombus.

### Revacept

**Figure 2:**
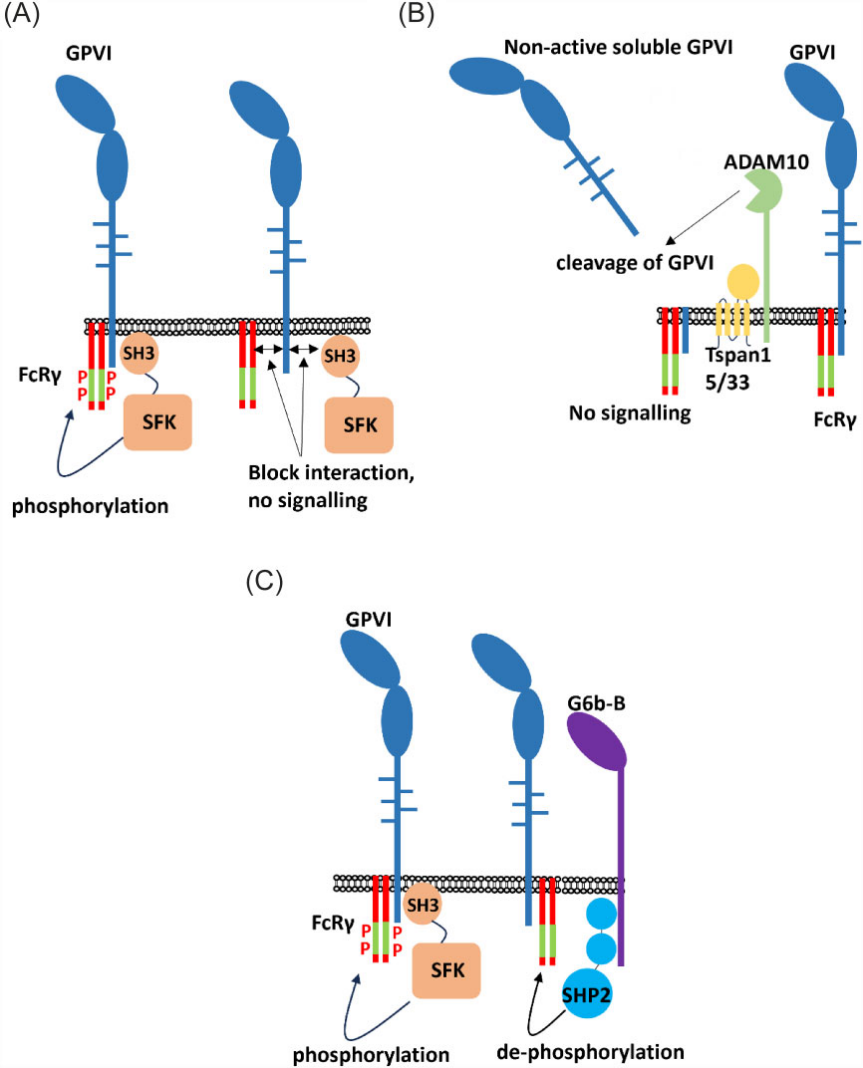
Alternative approaches to inhibiting GPVI signalling. (*A*) GPVI signalling requires the interaction of both SFKs and the FcR-γ chain to the intracellular tails of GPVI. SFKs phosphorylate the ITAM tyrosine residues of the FcR-γ chain upon GPVI activation, which leads to further downstream signalling. Blocking the interaction of GPVI with SFKs or the FcR-γ chain would lead to signal inhibition. (*B*) GPVI is cleaved by ADAM10 just before the transmembrane region in a process known as shedding. Extracellular GPVI is released from the platelet surface, leaving behind truncated GPVI consisting of transmembrane and intracellular region. This cleaved construct is no longer signal active due to it not being able to be clustered by ligands. ADAM10-induced shedding is mediated by Tspan5 and 33. (*C*) SFKs phosphorylate the ITAM sequences of the FcR-γ chain upon GPVI activation. This induces downstream signalling events. G6b-B associates with both GPVI and the intracellular phosphatase SHP2. Bringing SHP2 into proximity with the FcR-γ chain promotes dephosphorylation of the ITAM sequences and inhibits further downstream signalling. ADAM10, a disintegrin and metalloproteinase 10; GPVI, glycoprotein VI; ITAM, immunoreceptor tyrosine-based activation motif; SKFs, Src family kinases; Tspan, tetraspanin.

Revacept represents a different approach to GPVI inhibition compared with glenzocimab as it does not target platelets directly but binds to GPVI-specific ligands, thereby preventing them from binding to GPVI. Revacept is a recombinant dimeric form of extracellular GPVI fused to the human Fc fragment (*Figure 2C*). It consists of the entire extracellular region of GPVI including D1, D2, and stalk region. Revacept binds to collagen and prevents platelet-bound GPVI from binding to exposed collagen in atherosclerotic plaque.^70,87^ Revacept inhibits arterial thrombus formation in animal models, with no apparent increased risk of bleeding.^88–90^ In addition to collagen, revacept inhibits other GPVI ligands including vitronectin^91^ and fibronectin,^92^ but previous experiments have reported that revacept does not bind to fibrinogen and fibrin.^70^ Revacept has a relatively long half-life of over 5 days,^90^ but one potential drawback of revacept is its low potency when compared with antibody-derived ligands.^89^ Dimeric GPVI has an affinity of 576 nM,^61^ suggesting that the affinity of revacept for blocking collagen's interaction with GPVI is likely to be over 100-fold less potent than antibody-based inhibitors. This lower affinity equates to a higher dose being required for the same effects as an antibody-based inhibitor, although this is counteracted by the longer half-life resulting in a more long-lasting effect of the drug.

The Phase 1 trial for revacept was published in 2011 in which 30 healthy volunteers received intravenous administration of revacept of varying doses between 10 and 160 mg. Higher doses of revacept led to increased inhibition of platelet aggregation without drug-related adverse effects and no significant increase in bleeding time.^90^ A Phase II randomized clinical trial tested revacept vs. placebo in addition to standard antiplatelet therapy in patients with symptomatic carotid artery stenosis. In total, 158 patients were randomized to either placebo or 40 or 120 mg revacept. The primary efficacy endpoint for the trial was the number of new lesions detected on diffusion-weighted imaging cerebral MRI. Revacept did not significantly reduce the number of new lesions but also did not increase the risk of bleeding.^93^ Revacept has also been tested in a Phase II trial comprising 332 patients with stable coronary artery disease undergoing percutaneous coronary intervention. It was a double-blinded randomized trial in which patients received revacept 80 mg, revacept 160 mg, or placebo in addition to guideline-recommended dual antiplatelet therapy. The addition of revacept did not reduce the incidence of the primary endpoint (death or myocardial injury defined by a rise in troponin), and only the higher dose of revacept showed a significant, but small reduction in collagen-induced platelet aggregation. From a safety perspective, there was no increase in bleeding rate at 30 days compared with placebo.^94^ Overall, these neutral effects of revacept on the primary endpoint in these studies may be due to its relatively modest inhibition of GPVI combined with endpoints where GPVI has a relatively minor role.

## Other mechanisms that could be used to target GPVI

All reported direct GPVI inhibitors are thought to act by preventing ligand binding to the extracellular D1 and D2 domains. In addition to glenzocimab and revacept, there are many additional examples of inhibitors that target D1 and D2. These include Fab fragments JAQ1,^95^ OM2,^96^ and m-Fab-F.^65^ Single-domain antibodies, nanobodies, have also been documented to specifically inhibit GPVI binding to collagen.^64,73,97,98^ Due to their small size (∼15 kDa compared with 30 kDa for a Fab), the nanobodies do not induce the same steric effects exerted by the binding of larger antibody fragments, demonstrated by the fact that they inhibit collagen but not fibrinogen binding.^99^ A non-inhibitory nanobody has been used as a fluorescent probe to study the effect of GPVI inhibitors on thrombus formation.^98^ Finally, the small molecules honokiol and losartan have been shown to bind to the D1 domain and prevent clustering by collagen,^100^ but they have weak binding affinities and at high doses have been shown to induce off-target effects. Small molecules are not best suited for displacement of large multivalent ligands with multiple contact points but might be better suited for inhibiting other properties of GPVI.

Although there has now been success in targeting the extracellular domains of GPVI, there are other strategies that could be taken to effectively inhibit GPVI function. A critical step in GPVI activation is the phosphorylation of ITAM sequences on the GPVI-associated FcR-γ chain. The FcR-γ chain is critical for GPVI signalling,^95^ and blocking this interaction would result in complete inhibition of GPVI signalling. Key contact points within the intracellular tails could be targeted with membrane drugs (likely small molecules). However, no reagents have been targeted to the intracellular tails of GPVI and a better understanding for the structure of this region is required to design inhibitors. Similarly, the interaction of GPVI with SFKs would provide a similar approach to preventing the signalling machinery from associating with the receptor. ITAM phosphorylation results in the association and activation of Syk, which results in downstream signalling. Therefore, dephosphorylation of the FcR-γ could be an effective way of inhibiting GPVI. The challenge is how to achieve this in a specific manner. Receptors that contain immunoreceptor tyrosine-based inhibitory motifs (ITIMs) have the ability to dephosphorylate ITAM sequences through association with the phosphatases SHP1 and SHP2.^101^ Platelets express a range of ITIM-containing receptors including PECAM-1, G6b-B, and TREM (triggering receptor expressed on myeloid cells)-like transcript 1 (TLT1). Although surprisingly TREM1 activity has been linked with platelet activation,^102^ both PECAM-1 and G6b-B have been shown to inhibit GPVI-induced platelet signalling.^103,104^ ITIM receptors function by bringing active SHP1 and SHP2 phosphatases into close contact with ITAM receptors (*Figure 2B*). Agents that promote the specific association of GPVI with ITIM-containing receptors present a possible strategy for inhibition of GPVI signalling induced by any ligand of GPVI.

Another approach would be to remove GPVI from the surface. GPVI can be specifically removed from the surface of platelets through receptor shedding. This removal of GPVI is induced by shedding of the extracellular domain by metalloproteases.^105,106^ GPVI shedding has previously been shown to be induced by ligand binding including collagen, CRP, convulxin, and fibrin.^105,107^ GPVI shedding results in a soluble 55 kDa fragment consisting of the extracellular domains of GPVI and the remaining membrane-bound transmembrane and intracellular tail region (*Figure 2A*). The soluble GPVI portion can still bind to ligands but can no longer induce intracellular signalling. GPVI shedding is predominantly mediated by the metalloprotease a disintegrin and metalloproteinase (ADAM) 10.^108,109^ Cleavage of receptors by ADAM10 is mediated through the interaction of ADAM10 with tetraspanins (Tspans).^110^ It may be possible to take advantage of ligands that bind specifically to GPVI and induce shedding, but a greater understanding for the mechanisms of shedding is required.

## What next for GPVI inhibitors?

Recent early Phase II clinical trial results for GPVI inhibitors have shown promise. However, we will have to wait for larger clinical trials to determine the overall efficacy and safety of GPVI inhibitors. Due to the smaller sample sizes tested in Phase I and II studies, it is not yet possible to rule out clinically significant effects on haemostasis, although these have not been shown to date. Previously, vorapaxar, which is an antagonist of the protease-activated receptor 1 (PAR-1), was believed to be a promising antithrombotic inhibitor with no effect on bleeding.^111^ However, it was subsequently shown to increase the risk of intracranial haemorrhage.^112^ Reassuringly, glenzocimab has already been tested in the high-risk setting of stroke treated with thrombolysis, where intracranial haemorrhage is common, and there has been no sign of an increased risk of intracranial haemorrhage with potent GPVI inhibition.^24^

Our ability to target GPVI is narrowed by a lack of structural and mechanistic understanding for GPVI activation. As discussed in this review, there are many alternate methods, outside of inhibiting ligand-binding domains, that can be targeted or manipulated to inhibit GPVI function in response to all agonists. To fully take advantage of GPVI as an antithrombotic target, further studies are required that delve into the detailed mechanisms of GPVI function spanning from extracellular and intracellular events to interactions with other proteins and receptors in the membrane.

## Data availability

No new data were generated or analysed in support of this research.
